# Single-step controlled synthesis of flower-like gold nanoparticles stabilized by chitosan for sensitive detection of heparin using a surface-enhanced Raman scattering method†

**DOI:** 10.1039/d2ra06528b

**Published:** 2022-12-06

**Authors:** Thu Anh Nguyen, Anh Nguyen Kim Do, Tien Nu Hoang Lo, In Park, Khuong Quoc Vo

**Affiliations:** Faculty of Chemistry, University of Science, Vietnam National University – Ho Chi Minh City 227 Nguyen Van Cu Street, Ward 4, District 5 Ho Chi Minh City 70000 Vietnam vqkhuong@hcmus.edu.vn; Research Institute of Clean Manufacturing System, Korea Institute of Industrial Technology (KITECH) 89 Yangdaegiro-Gil, Ipjang-myeon Cheonan 31056 South Korea inpark@kitech.re.kr; KITECH School, University of Science and Technology (UST) 176 Gajeong-dong, Yuseong-gu Daejeon 34113 South Korea

## Abstract

A novel single-step and template-free procedure, including controlled synthesis of gold flowers (AuNFs), conjugation to a 4-MBA reporter, and stabilization with chitosan, is proposed to develop the SERS tags-based nanoparticles for trace detection of heparin. This SERS detection assay is based on the aggregation/non-aggregation balance of AuNFs-4-MBA@chitosan nanoparticles, which was induced by adding a very low concentration of heparin in the as-synthesized colloidal solutions. SERS-tag colloids are prepared by mixing chitosan with HAuCl_4_ and 4-mercapto benzoic acid before being reduced with ascorbic acid under appropriate pH conditions. The formed AuNFs-4-MBA@chitosan nanoparticles were positively charged with high stability and well-dispersed in aqueous media. Based on understanding each reaction component's role in the preparation of the SERS tag colloid, we aim to simplify the controlled synthesis and Raman probe conjugation process. The average size of AuNFs is below 90 nm, fine-tuned in shape and effectively conjugated to the Raman reporter molecules 4-MBA. These as-prepared SERS tag-based AuNFs have good biocompatibility and are virtually non-toxic, as studied with fibroblast and MCF-7 cells. Through these SERS-tag colloids, the trace detection of heparin is improved, with a wide detection window (0.01 to 100 ppm), high reproducibility (RSD value of 3.56%), limit of detection (LOD) of 0.054 ppm, and limit of quantification (LOQ) of 0.17 ppm. Comparison experiments show that the SERS-tag colloids possess good selectivity over other ions, and organic and amino acid substances. The results provide the capability and the potential for application under complex biological conditions and future biosensing based on SERS signal amplification.

## Abstract 2

A novel single-step and template-free procedure is proposed to develop the SERS tags-based nanoparticles for trace detection of heparin.

## Graphical abstract

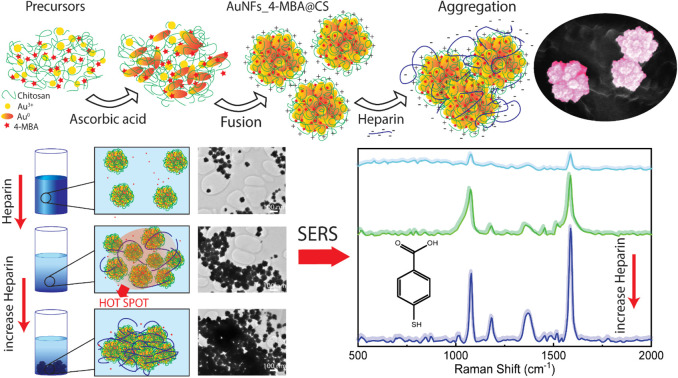

## Introduction

The trace determination of specific biomolecules for clinical treatment with high selectivity and sensitivity is essential for application in future diagnosis research. Among the therapeutic drugs derived from bio-molecules, heparin has long been used clinically as an anticoagulant in cardiovascular surgery or long-term therapy.^1^ However, misuse of heparin therapeutic doses in surgery or during the postoperative period could cause adverse effects such as allergic reactions,^2,3^ hemorrhages,^4^ and thrombocytopenia.^5^ Heparin comprises 1-4-linked pyranose-uronic acid and 2-amino-2-deoxy-glucopyranose residues that regulate biological processes such as immune defense and coagulation.^6,7^ Heparin is a typical negatively-charged linear polysaccharide,^8,9^ which could induce electrostatic interaction with various proteins,^10^ growth factors,^7^ and lipoproteins.^11^ Many methods have been proposed in the literature for determining heparin, such as fluorescent sensors,^12^ colorimetric probes,^13^ and direct naked-eye detection.^3^ However, these methods have not been able to fulfill all the expectations of modern bio-molecule analysis cause of the shortcoming of sensitivity,^3^ being far away from the biological environment, or long time-consuming.^14^ Among the various methods developed for heparin analysis, surface-enhanced Raman scattering (SERS) is a unique spectroscopy technique that has extraordinary potential in the analytical field of organic compound trace determination. These intrinsic spectra can provide structural information to enhance single-molecule detection with extreme sensitivity.^15^ In recent years, strategies of Raman signal amplification have been developed based on the intersections space of two or more neighboring noble metal nanoparticles that are supposed to generate the strongest electromagnetic fields.^16,17^ When the analyte molecules enter these intersection spaces, termed “hot spots”, the intensity of the Raman signals significantly arises. Another strategy for trace determination is based on the signal change of the reporter molecules conjugated on the surface of the nanoparticles,^18^ which mainly depends on noble nanoparticle aggregation and non-aggregation balance. The indirect methods can be utilized in biological sensing applications, especially biochemistry and life sciences.^19^ Raman peak intensity could be optionally controlled by manipulating the self-aggregation degree of the formed particles in colloidal solution with the presence of reporters. The advantages of this novel strategy are that it can detect complicated and high molecule weight organic compounds with many functional groups, including protein,^20^ enzyme, or DNA,^21^ in very small trace amounts. Wu *et al.* proposed a “turn-off” SERS biosensor for thrombin with the 4-mercaptobenzoic acid (4-MBA) conjugated gold nanoparticles. Arginine peptides were used to induce gold nanoparticle aggregate, and thrombin was employed to segregate the nanoparticles conjugated with the reporter. This strategy can remarkably improve the sensitivity with the limit of detection of 160 fM.^22^

Gold nanoparticles (AuNPs) with high order complexities are widely used as the nano-substrate for developing SERS tags due to many outstanding advantages such as the local optical fields at their surfaces,^23,24^ facile synthesis,^25^ unique characteristics,^26,27^ and high biocompatibility.^28,29^ The Raman signal amplification ability of gold nanoparticles is strongly influenced by their sizes,^30^ geometric morphologies,^31–33^ and aggregation form.^34,35^ Among many nanostructures, flower-liked gold nanoparticles are compelling for emerging applications in the biochemical field with the increase in the effective conjugation of bio-molecules due to their unique morphology.^36,37^ Additionally, gold nanoflowers (AuNFs) with exceptional optical properties can be exploited in the trace determination of biological compounds related to their aggregation state. The aggregation and non-aggregation relate to the internal distance, dielectric properties, size, and shape of nanoparticles and the environment. The aggregation of metal nanoclusters can enhance the intense electromagnetic field at the connections between nanoparticles, known as “hot spots”.^38,39^ The intensity of the Raman signal could arise due to the movement or adsorption of molecules in these active “hot spots”.^40^ Precise tuning of the aggregated nanoparticle morphology is thus vital in designing nanoprobes for SERS. However, preparing bare AuNPs SERS tags with the exposed surface is easily compromised by the dissociation of the reporters or the adsorption of the interfering molecules in the biological environment. Various protecting agents, such as polymers,^41,42^ liposomes,^43,44^ silica,^45^ and biomolecules,^46^ were used to encapsulate the SERS tags to overcome these limitations. These agents can attach to gold nanoparticle surfaces due to the chemisorption, electrostatic and hydrophobic interaction.^25^ Chitosan (2-amino-2-deoxy-*b*-β-glucan) is widely used among many biodegradable molecular compounds because of its ability to simultaneously reduce and stabilize the gold nanoparticles based on their surface-active properties. Furthermore, the most significant advantages of using chitosan are high biocompatibility^47^ and non-toxicity^9^ for extensive application in medical fields. As a polysaccharide polymer with highly functional groups and a flexible structure, chitosan can be used to replace some soft-template agents in controlling the preferential growth of the seeds to form AuNFs. Besides the structure-directing role, chitosan could also be exploited to immobilize bio-molecules for developing nanomaterials applied in protein detection.^9,48^ Although many studies on synthesizing gold nanoparticles with chitosan were reported, very few works proposed methods for combining controlled synthesis of AuNFs and conjugated SERS reporters in a single-step process. For example, Sun *et al.* described a one-pot synthesis of AuNPs using chitosan with different degrees of deacetylation and molecular weight.^49^ Phan *et al.* recently developed an efficient preparation route of gold nanostars for photo-thermal therapy and photoacoustic imaging with chitosan.^47^ Franconetti *et al.* reported a theoretical method based on Dissipative Particle Dynamic (DPD) to reveal steric and electro-steric mechanisms to understand the interaction between chitosan and gold nanoparticles.^50^ Wang *et al.* proposed a facile preparation method to fabricate flower-like gold nanostructures with the presence of luminol and chitosan.^51^

**Fig. 1:**
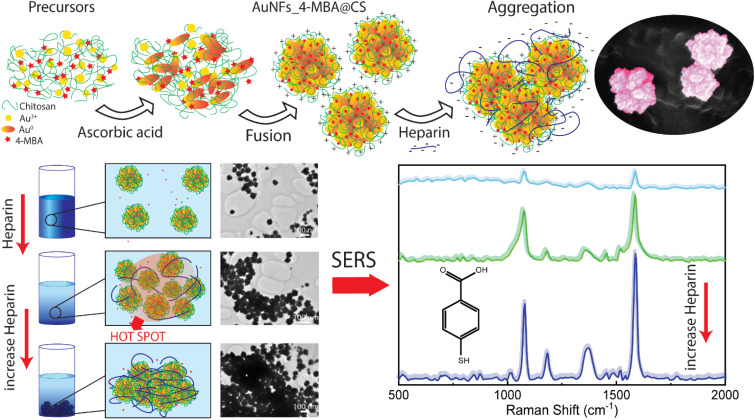
Scheme for preparation of AuNFs-4MBA-CS colloidal solution with the controlled particles sizes and SERS detection of heparin through the inducing aggregation effects.

This work developed the novel method of simultaneously combining the controlled synthesis of AuNFs, conjugated with Raman reporter 4-mercaptobenzoic acid (4-MBA) and encapsulated by chitosan in a single-step process. These SERS tags based on anisotropic nanoparticles were designed by manipulating the stabilizer concentration and pH. The signal improvement of SERS reporter conjugated on the nanoparticles can be achieved through the presence of many hot spots, which are generated from the interstitial voids created by small branches of the neighboring AuNFs, and on the rough surface of a single particle (Fig. 1). The use of chitosan possesses three key advantages: structure directing the formation of the flower-liked particles, enhancing the stability and biocompatibility of the SERS-tags, and increasing the self-aggregation of the SERS tags for amplifying the 4-MBA signal by adding the analyte molecules. For the trace detection of heparin, an aggregation mode of the SERS platform was conducted in a controllable manner, based on the electrostatic interaction between negatively charged heparin and positively charged chitosan under appropriate conditions. The addition of different heparin concentrations induces AuNFs-4-MBA aggregation, leading to the enrichment of the hot spots on the AuNFs surface; thus, the intensity of characteristic Raman peaks was significantly amplified. Besides, the bio-compatibility of as-prepared AuNFs-4-MBA-CS was evaluated with the cell viability of fibroblast and MCF-7 using sulforhodamine assay.

## Experimental

### Chemical and materials

Tetrachloroauric (III) acid trihydrate (HAuCl_4_·3H_2_O, 99.9%), acetic acid (CH_3_COOH, 99.5%), and ascorbic acid (C_6_H_8_O_6_, 99.7%) were purchased from Merck (Darmstadt, Germany). Chitosan (CS, (C_12_H_24_N_2_O_9_)_*n*_, >75% deacetylated, *M*_w_ 310 000–375 000 Dalton) was purchased from Sigma-Aldrich (St. Louis, MO, USA), and used without post-modification. Collagenase Type 1A, fetal bovine serum (FBS), l-glutamine, (4-(2-hydroxyethyl) piperazine-1-ethane sulfonic acid) (HEPES. 99.5%), anti-vimentin/anti-cytokeratin 19 anti-bodies, amphotericin B, penicillin G, and streptomycin were obtained from Sigma-Aldrich (St. Louis, Missouri, USA). Gibco Dulbecco's Modified Eagle Medium: nutrient mixture F12 (DMEM/F12) was purchased from Thermo Fisher Scientific (Waltham, MA, USA).

Chemicals are analytical grade and used without further purification, and the deionized water (conductivity below 4.3 μS cm^−1^) was used in all the aqueous phase experiments.

### Preparation of SERS tags based on gold nanoflowers

AuNFs were prepared under mild condition by reducing tetrachloroauric (III) acid trihydrate with ascorbic acid in the chitosan (CS) aqueous solution to create surfactant-free gold nanoparticles. Before synthesis, all glassware were cleaned with aqua regia (HNO_3_ : HCl, 1 : 3, v/v) and rinsed again with deionized water. For preparing CS with concentration range, a different amount of CS powder was dissolved into an acetic acid aqueous solution (1.0%, v/v). The obtained mixture was magnetically stirred at 800 rpm at 60 °C under the pH condition of 4.0 for four hours to complete the solubilization of the powder.

In a single-step process, an aqueous HAuCl_4_ (2.5 mM, 2.0 mL) solution was previously mixed with a 1.0 mL volume of 10 mM MBA, role as SERS reporter, and then added into CS solution under appropriate pH conditions (varying from 1.0 to 8.0) while gently stirring for 5 min at the ambient temperature. The pH was adjusted by adding different amounts of 0.10 M NaOH and 0.1 M HCl solution, followed by magnetically stirring for 2.0 min. Subsequently, 0.50 mL ascorbic acid 0.10 M was added dropwise to the above solution, magnetically stirring at 600 rpm for 5 min. The total volume was about 20.0 mL by adjusting with DI water, and the colloidal solution was then left undisturbed for nine hours at room temperature to complete the reaction and conjugation process. The colloidal solutions changed colour slowly from transparent yellow to red, purple-red, and dark-blue; the samples were then stored at 4.0 °C. The above mixture was centrifuged at the rate of 6000 rpm for 20 minutes to remove the excess unbound 4-MBA. The supernatant fraction was discarded, and the obtained sediment was re-dispersed in 20.0 mL DI water.

### Cell lines and cell culture

MCF-7 was obtained from the American Type Culture Collection (Manassas, Rockville). Cells were cultured at 37.0 °C and 5.0% CO_2_ in Eagle's Minimal Essential Medium (EMEM), supplemented with 10% (v/v) FBS, 2.0 mM l-glutamine 20.0 mM HEPES, 0.025 μg mL^−1^ amphotericin B, 100 IU mL^−1^ penicillin G and 100 μg mL^−1^ streptomycin.

### Fibroblast culture

The method was conducted based on the previously reported by Takashima with some modifications.^52^ Typically, the dermal sample was cut into small (2–3 mm × 5 mm) squares with the fine surgical scalpel. Skin pieces were placed in the center of a tissue culture dish and incubated with 0.2% (w/v) collagenase type IA in PBS at 37 °C for 2 hours. A cell strainer with 70 μm mesh was used to filter the cell suspension. The fibroblast suspension was centrifugated at 200×*g* at 4 °C for 10 min. The cell pellets were resuspended in Dulbecco's Modified Eagle Medium: nutrient mixture F12 (DMEM/F12). Culture fibroblast cells in DMEM/F12 supplemented with 10% (v/v) FBS, 20 mM HEPES, 0.025 μg mL^−1^ amphotericin B, 100 IU mL^−1^ penicillin G, 100 μg mL^−1^ streptomycin at 37 °C, 5% CO_2_.

### Sulforhodamine B (SRB) assay

The SRB assay was applied for the cytotoxicity determination based on the measurement of cellular protein content. Cells at a culture density of 7500 cells per well were inoculated for 24 hours. The AuNFs-MBA-4@CS were added at different concentrations for the next 48 hours. The total protein content of treated cells was preserved constantly for 1–3 hours by using the cold trichloroacetic acid solution at 50% (w/v) concentration. After an incubation period, the cells were stained with the 0.2% (w/v) sulforhodamine B solution for 20 minutes and washed five times with the 1.0% acetic acid. The protein-bound dye was then dissolved in a 10 mM Tris base solution. The absorbance measurement at two wavelengths of 492 and 620 nm was conducted on a 96-well microtiter plate reader (Synergy HT, Biotek Instruments). Each experiment was repeated three times, and the results were performed as mean ± standard deviation.

### Data analysis

After obtaining the optical density value at the wavelength of 492 or 620 nm (denoted as OD_492_ or OD_620_).

1 Calculate the value of OD_492_ (or OD_620_) = OD_averg_ − OD_blank_

Calculate the percentage (%) of cytotoxicity according to the equation:2
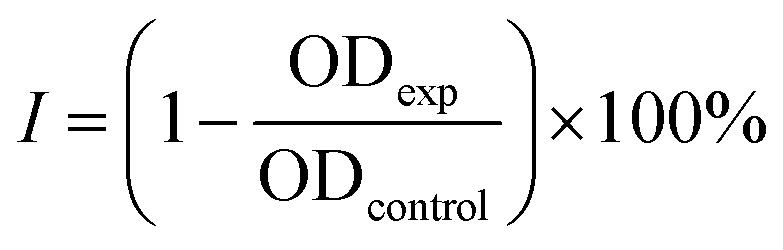
which OD_averg_: OD value of the well-containing cells. OD_blank_: OD value of blank well (no cells). OD_exp_: OD value of the testing samples from eqn (1). OD_control:_ OD value of the control determined from eqn (1).

### Sample preparation for detection of heparin based on AuNFs-4-MBA@CS SERS-tags

The as-prepared SERS-tag colloidal solutions were used for the heparin detection based on the aggregation effect of the formed AuNFs induced by adding heparin solution. In a typical procedure, 40 μL of heparin solution with different concentrations was added to 360 μL of freshly prepared AuNFs-4-MBA@CS and left undisturbed for 5 min at room temperature before being analyzed with the Raman technique.

### SERS measurements procedure

The SERS analysis was carried out with or without heparin using the XploRa Plus confocal Raman Microscope (Horiba SAS, Longjumeau, France). An excitation laser source was 532 nm and operated at 50 mW of power. LabSpec 6 software suite (Horiba France SAS) was used to analyze the Raman spectra.

### Characterization

The UV-Vis absorbance spectroscopy of prepared AuNFs-chitosan colloids was characterized on a UV-Vis-NIR-V670 spectrophotometer (JASCO International Co. Ltd, Tokyo, Japan), using a quartz cuvette of 1 cm path length at room temperature and measured from 300 and 800 nm of wavelength at the scanning rate of 200 nm per minute. FTIR spectra were recorded as KBr pellets using Bruker Equinox 55 FTIR spectrometer (Agilent Instruments Co. Ltd, Frankfurt, Germany) in 400–4000 cm^−1^. The samples were prepared with potassium bromide (KBr) at a 2.0–5.0% ratio and compressed for analyzing the spectrum further. The XRD pattern was determined using D8 Advance-Bruker (Agilent Instruments Co. Ltd, Frankfurt, Germany) using Cu-Kα radiation in the 2*θ* range between the 20° to 80° (scanning rate of 4° per minute and operated at 40 kV). A JEM-1400 Transmission Electron Microscope (TEM) (JEOL Ltd., Tokyo, Japan) was used to observe the morphologies and size of AuNFs. Drops of AuNFs colloidal solution were placed onto a carbon-coated copper grid (300-mesh, Ted Pella Inc., USA) to prepare the TEM analysis sample, followed by evaporation for 15 minutes the TEM images were obtained at an acceleration voltage of 200 kV). The average geometric diameter and size distribution of AuNFs were statistically estimated with the mean value of 300 particles in several selected microphotograph areas. The dynamic light scattering (DLS) measurements were performed using an SZ-100 (Horiba, Instrument Co. Ltd, Kyoto, Japan) with a sample container cuvette (volume of 100 μL). The Z-average and the mean size of AuNFs have measured three replicates for each sample. The zeta potential measurements of chitosan-coated AuNFs were conducted at 25 °C using a Zetasizer SZ-100 (Horiba, Instrument Co. Ltd, Kyoto, Japan). The 1.0 mL aliquot sample was added to 2.5 mL polystyrene disposable cuvettes with a path-length of 1.0 cm. The samples were measured with an equilibrium time of 150 seconds at 25 °C. Three repeated measurements were carried out to calculate the average zeta potential values. The solution pH was measured using the pH-71 Meter (Horiba, Instrument Co. Ltd, Kyoto). An X-ray photoelectron spectroscopy (XPS; Thermo Scientific, Waltham, MA, USA), using a monochromatic Al Kα X-ray source, was used to investigate the compositions of the AuNF-chitosan samples at the photon energy of 1486.7 eV.

## Results and discussion

### Controlled size of formed gold nanoflowers by varying chitosan concentration (AuNFs)

**Fig. 2:**
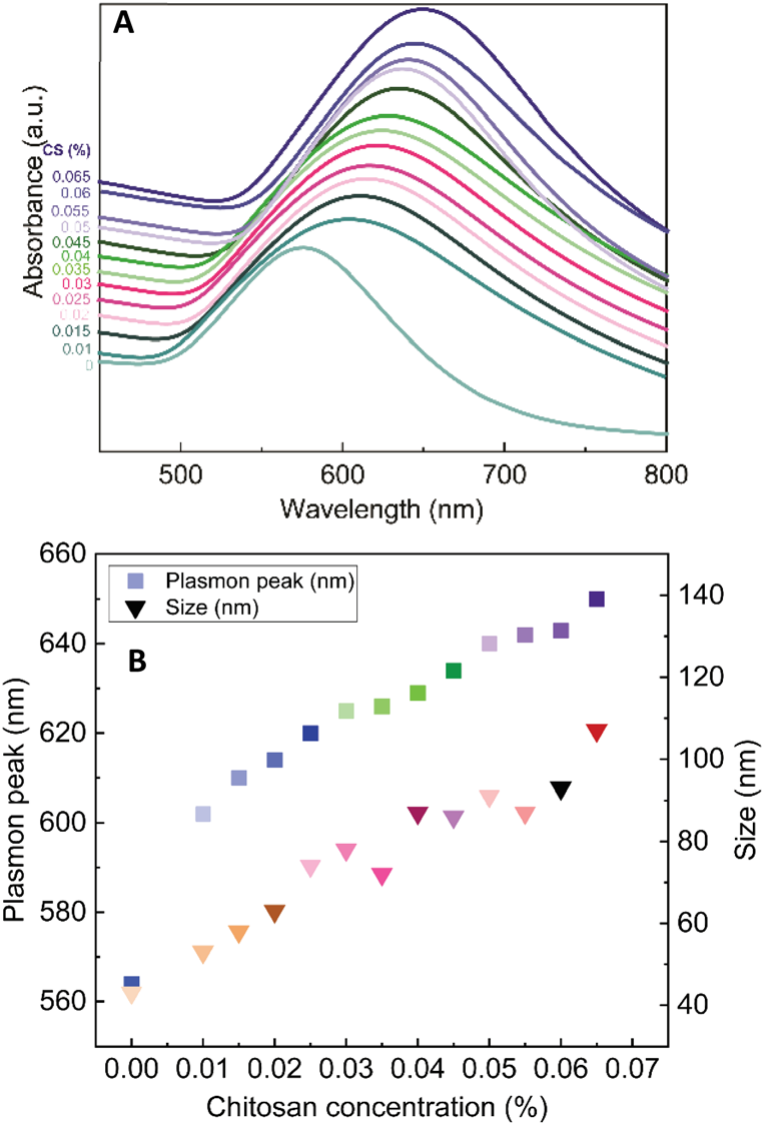
(A) UV-Vis absorbance spectrum of AuNFs synthesized with 5.0 mL of different chitosan concentrations ranging from 0% to 0.065% at pH 4.0 in aqueous solutions. (B) Comparison of the particles size measured by SEM and plasmon peaks.

**Fig. 3:**
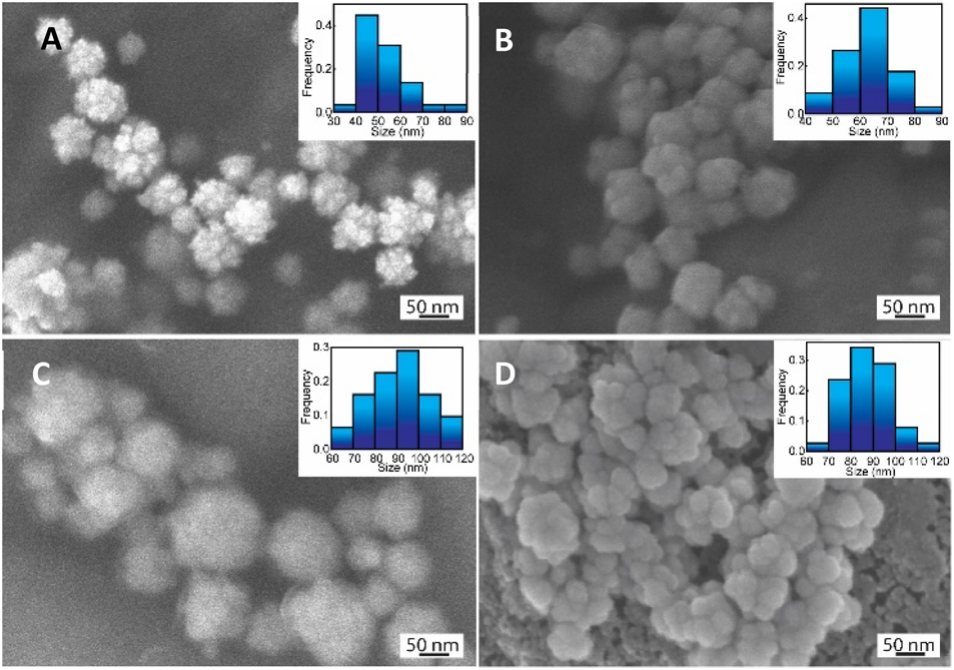
SEM images and corresponding histogram (right-inset) of flower-like gold nanoparticles prepared with different chitosan concentration (A) 0.01, (B) 0.03, (C) 0.05, (D) 0.06, (%, w/v).

The role of CS as the structure-directing and protecting agent for the controlled synthesis of AuNFs was investigated through the compared experiment conducted without using CS. As shown in Fig. 2, only one weak and narrowband located at 578 nm with low intensity is observed in the spectrum of the gold nanoparticles sample synthesized in the absence of CS. This band could be ascribed to the surface plasmon resonance (SPR) of spherical shape in the colloidal solutions.^53^ When synthesized with CS, a remarkable red shift of the SPR band from 578 to 614 nm was observed (Fig. 2), corresponding to the increase of CS concentration from 0 to 0.01%, respectively. This change suggests that anisotropic nanoparticles could be formed instead of spherical ones when prepared with the presence of CS. Based on previous reports, the SPR band red-shifts significantly to a longer wavelength from the band of spherical morphology could be due to the plasmon resonance of the anisotropic nanoparticles.^54–56^ Besides the red-shift of maximum wavelength, adding more CS (from the concentration of 0.01 to 0.065%) leads to a broader plasmonic band and increased SPR intensity. It can be interpreted that the productivity of formed nanoparticles increased with the increase of CS concentration. SEM observation subsequently evidenced this point; Fig. 3A–D exhibits the corresponding SEM images of AuNFs with increased CS concentration from 0.01 to 0.06 (%, w/v). The flower-like nanostructures consist of many smaller particles (Fig. 3A). These nanodots approach each other and begin to self-aggregate through fusing by oriented attachment to form larger-sized AuNFs. Besides, the presence of ascorbic acid can primarily facilitate the generation of nanodots, which induce the self-aggregation to form AuNFs. The lower-level contrast substances covered around AuNFs indicated that the CS layer had been adsorbed on the surface of AuNFs-4-MBA (Fig. 3C). At such high concentrations, the functional groups in CS could have more chances of binding on AuCl_4_^−^ ions, forming more AuNPs. The intensity increased, accompanied by a continued concentration increase from 0.01% to 0.06% (w/v), and the SRP band also shifted to a longer wavelength (from 614 to 649 nm). However, when it reached a higher concentration (0.07%), the colloidal sample was unstable and flocculated for one day of storage. It implied that the size, shape, and productivity of AuNFs depended on the critical concentration of CS. When exceeded the critical concentration, the polymeric CS chains could become entangled or aggregated due to the inter hydrogen bonding.^57,58^ Additionally, CS used in this synthesis has a significant molecular weight (310 000–375 000 Dalton), making the molecules easy to entangle at high concentrations.

**Fig. 4:**
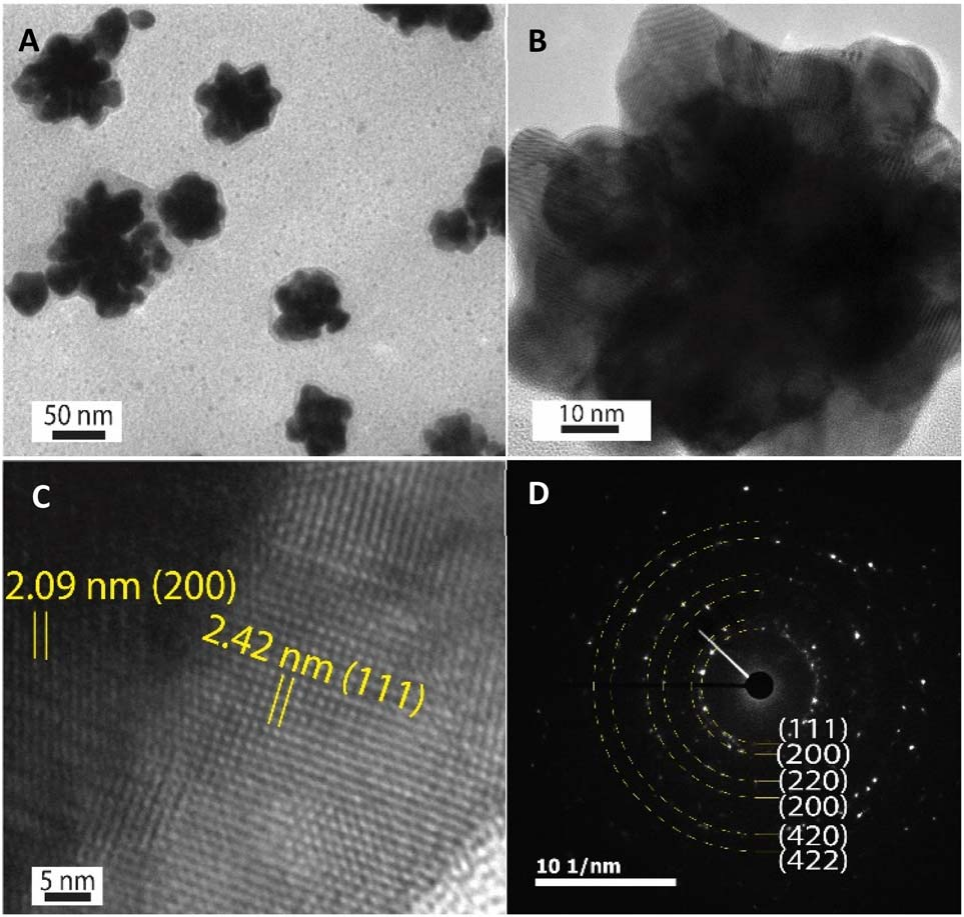
(A) TEM images of AuNFs prepared with the chitosan concentrations of 0.06% (v/v) (scale bar, 50 nm), (B) high magnification TEM images of one AuNF particles (scale bar, 10 nm), (C) HR-TEM images of one AuNFs consists of *d*-spacing of Au fcc crystalline-structure (scale bar, 5 nm), and (D) corresponding SAED pattern of the AuNFs-4-MBA@CS.

The HR-TEM technique was further used to investigate the morphology of one Au nanoparticle. According to HR-TEM images presented in Fig. 4A–C, it is clearly shown that one AuNFs was formed from many quasi-spherical particles. The lower-level contrast shell adsorbed around the particles was also observed in Fig. 4A, indicated that the presence of chitosan on the AuNFs surfaces. The lattice spacing of gold crystal is shown in Fig. 4C with the *d*-spacing value of 2.42 Å, which contributed to the (111) plane.^56^Fig. 4D displayed the selective area electron diffraction (SAED) pattern with typically blurred diffraction rings. The innermost diffraction ring corresponds to (111) planes; the rings to the outside are (200), (220), and (311) planes. The polycrystalline materials composed of small nanoparticles often form ring patterns.^59^ The high-resolution TEM image in Fig. 4B confirms this point; the flower-like gold nanoparticle was formed with many smaller particles. Based on these analysis results, the growth AuNFs is predominantly due to the self-assembly process. However, the structure-directing role of CS in the controlled synthesis of AuNFs has not been precisely explained. It is believed that it might be relevant to the preferential adsorption of the –NH_2_ functional group on specific planes of the initial Au seed,^60^ and the ability to limit the growth space of small-sized nanoparticles due to the length of the chitosan chain. These effects could slow down the fusion process of crystals along the specific planes and thus might promote other directions, eventually forming the flower-like nanoparticles with controlled sizes. Accordingly, an appropriate CS concentration is vital for the controlled synthesis of AuNFs. It seems that could provide a sufficient –NH_2_ amount for binding to specific crystal faces of existing Au seed particles.

### Varying the pH value

**Fig. 5:**
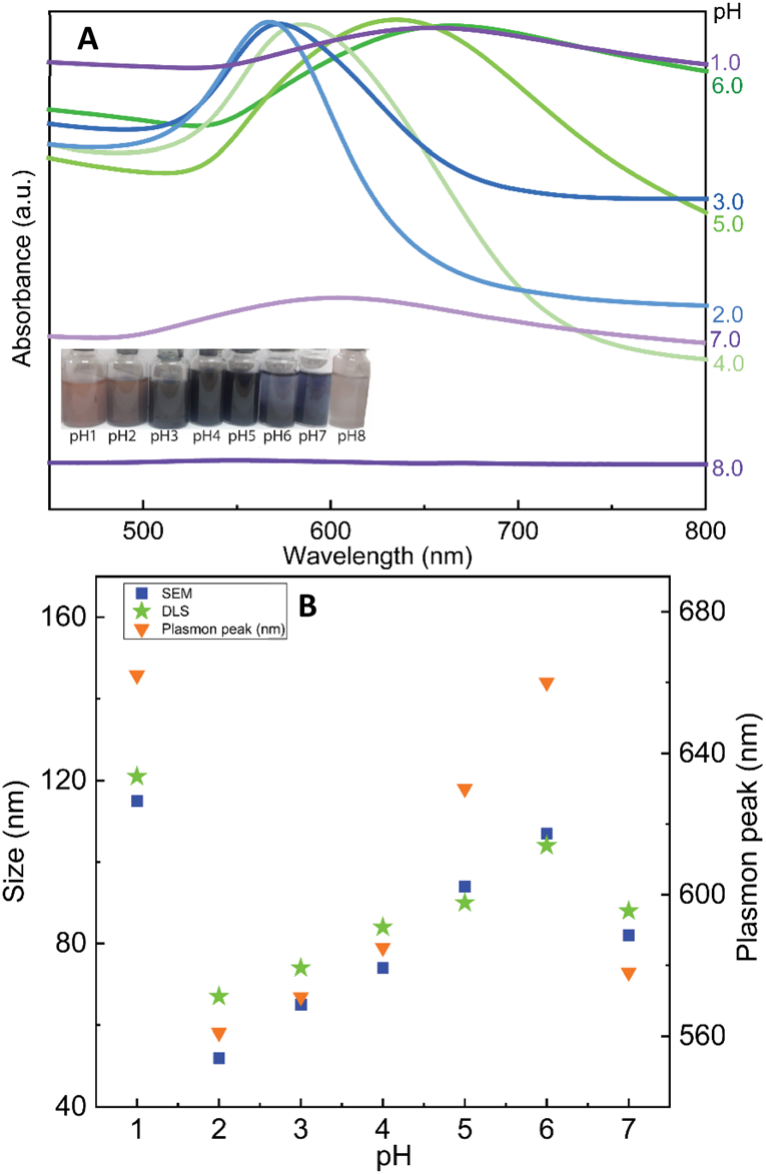
(A) UV-Vis absorbance spectrum of AuNFs synthesized with 5.0 mL chitosan 0.06% at different pH conditions ranging from 1.0 to 8.0 in aqueous solution, and (B) comparison of the particles size measured by SEM and the plasmon peaks.

Herein, we discovered that the change of pH condition is responsible for the binding force of CS to the surface of Au nanoparticles. It can be hypothesized that the formation of nanoflowers is based on the loose contact points of CS and Au. For studying the effect of pH on the formation of AuNFs by using CS as a structure-directing agent, eight experiments in a range of pH conditions from 1.0 to 8.0 were performed with the fixed CS concentration of 0.05% (w/v). As shown in Fig. 5A (left inset), the colour of dispersion changed from transparent blue to bright blue and then dark blue, when varied pH value range 1.0–4.0. Moreover, the absorbance peaks undergo a remarkable red shift from 582 to 658 nm by changing the pH values from 4.0 to 7.0. These changing features could be attributed to the formation of the AuNFs, the adsorption bands located around 627 to 660 nm relevant to the in-plane dipole plasmon resonances of nanoparticle tips.^54,61^ Furthermore, this red-shift of the surface plasmon resonance peak indicates the formation of larger-sized particles, which is confirmed by the DLS results (83 nm, 92 nm, and 122 nm corresponding to pH of 4, 5, and 1, respectively) (Fig. 5B).

**Fig. 6:**
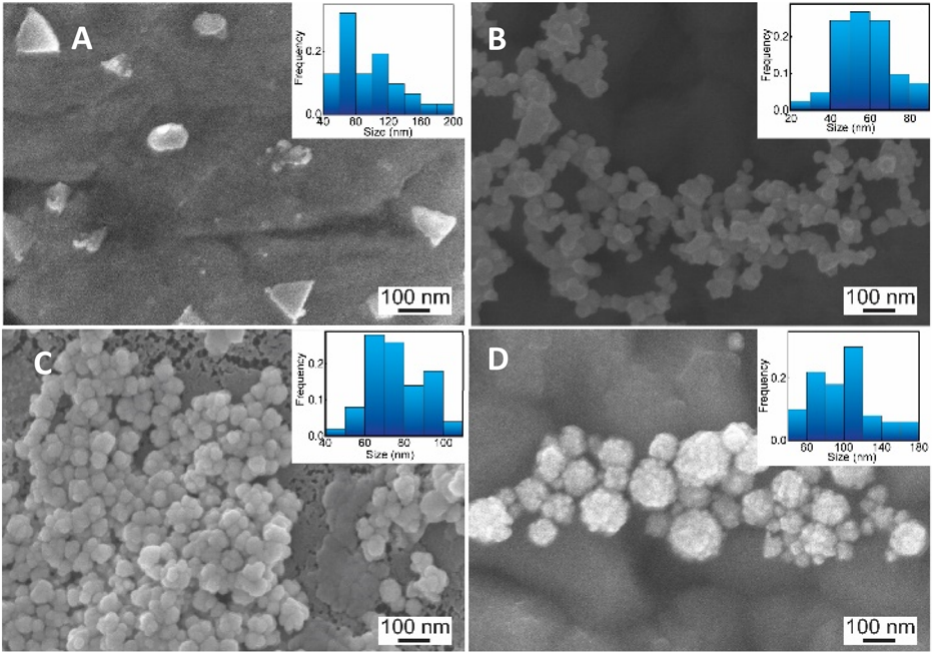
SEM images and corresponding histogram (right-inset) of flower-like gold nanoparticles prepared with chitosan at different pH conditions: (A) pH 1.0, (B) 2.0, (C) 4.0, and (D) 6.0 (scale bars for all, 100 nm).

Relying on the previous literature, the hydrolysis of AuCl_4_^−^ occurs at pH 3.0–4.0, and ions Cl^−^ are partially substituted by hydroxide ions (exists in small amounts in weakly acidic environments) to evolve into AuCl_(4−*x*)_OH_(*x*)_ form.^62^ It can lead to a slight decrement of the redox potential of Au(iii) complexes which slows down the rate of the gold reduction reaction.^63^ Then, the existing nuclei are predominantly fused due to the adsorption of the formed Au atom instead of creating newer nuclei, which may explain the formation of flower-liked morphology. However, it is worth noting that the rate of reaction between the gold salt and ascorbic acid is fast, so in this case, the interaction between CS and Au(iii) complex would play a key role in controlling the size and shape of AuNPs.^63^ Electrostatic interaction between positively charged ammonium of glucosamine moieties and the negatively charged AuCl_4_^−^ can be effective at a pH of above 3.5.^55^ At the pH reaction medium of 4.0, the amino groups of CS stay at their protonate, forming the ammonium group (–NH_3_^+^), thus leading to the effective interaction of the Au(iii) complex. The binding of the glucosamine unit can be formed with the replacement of chloride atoms in the Au(iii) complex by –NH_3_^+^ groups.^62,64^ Subsequently, the Au(iii) binding ions were reduced by ascorbic acid to generate Au^o^, which further fused to the initial seed to create AuNFs. In this condition, the growth of AuNFs is governed by the template effect of the CS chain and the amount of Au(iii) ions that bind to glucosamine units. According to this theoretical point of view, the morphology of AuNFs is limited by the enclosed space created by the CS coverage layer. Thus the particles with flower-like morphologies might be predominantly formed. The FE-SEM results support the influence of pH on the shape and size of AuNFs. Fig. 6A–D presents the microscopy images of as-prepared AuNFs samples in different pH conditions. Several types of nanoplates with triangular and irregular shapes are observed in the SEM image of the Au sample prepared at pH 1.0 (Fig. 6A) with an approximate size distribution between 80 and 100 nm. Consistent with the UV-Vis spectrum of AuNPs prepared at pH 2.0, it is observed that the main products are flower-like shape particles composed of small spherical nanoparticles (Fig. 6B). Specifically, as shown in Fig. 6C and D, the AuNFs were formed in the large numbers at the pH range of 4.0 to 6.0.^65^ For DLS measurements, the decrement of average sizes from 108 nm to 84 nm is observed when increased pH conditions from 6.0 to 7.0 (Fig. 5B). Energy dispersive spectroscopy (EDS) of formed AuNFs-4-MBA@CS was used to characterize the composition of elements in the samples. Furthermore, the morphologies of particles were also confirmed by SEM (see Fig. S1†). The strong intensity peaks observed at 2.1 and 2.3 eV are assigned to the signal of the Au element. Peaks corresponding to S, C, O, and Cl elements also appeared on the EDS spectrum (see Fig. S1A†). However, the atoms percentage is relatively low, which partially reflects the presence of the 4-MBA Raman probe and chitosan molecules on the surface of AuNFs.

The chemical composition and atomic ratios of the AuNFs@CS were identified through the XPS method (see Fig. S2†). The survey spectra showed the Au, C, N, and O elements. The N_1s_ spectra of the AuNFs-CS showed the presence of a peak intensity of 400.0 eV, which was attributed to the chitosan amine group (NH_2_).^66^ Two peaks were obtained in the high-resolution N_1s_ spectrum of AuNFs-CS located at 400 and 402 eV assigned to the protonated amine groups of CS.^67^ The presence of protonated amino groups indicates the predominant amine sites for interacting with Au nanoparticles. This interaction was electrostatic between the positively charged amino group of CS and the negative charge on the surface of Au nanoparticles. The peak at 402 eV on the N_1s_ spectrum only appeared in the AuNFs-CS sample could evident the interaction between the AuNFs and chitosan.

### X-ray diffraction of AuNFs-CS

**Fig. 7:**
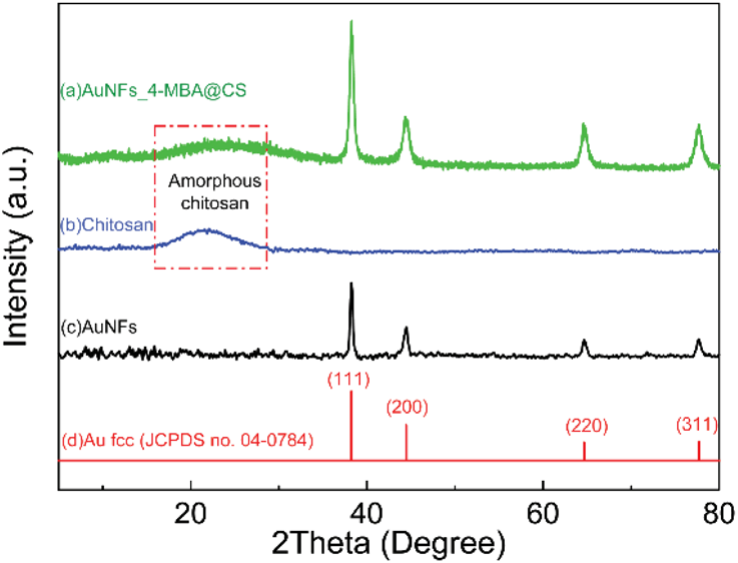
X-ray diffraction pattern of (a) AuNFs-4-MBA@CS with the concentration of 0.06% (w/v), (b) pure chitosan, (c) AuNFs prepared without the presence of chitosan, and (d) standard XRD pattern of Au fcc crystalline structure (JCPDS no. 04–0784).

X-ray diffraction is a well-determined method to characterize the crystalline structure, and it gives information about the formed nanoparticles depending on their size. From the XRD pattern of AuNFs-4-MBA@CS presented in Fig. 7A, there are four diffraction peaks centered at 2*θ* of 38.1°, 44.2°, 64.2°, and 77.1°, which correspond to the (111), (200), (220), and (311) lattice planes of face-centered cubic (fcc) of gold crystalline structure, respectively.^47,60^ These diffraction peaks are relatively broad due to the small particles size. In addition, a diffraction peak attributed to the (111) lattice plane has stronger intensity than others, indicating that the (111) lattice plane should be the basal plane. The XRD data were thus commensurate with the previous studies that the gold AuNSs were formed as pure gold nanocrystal.^47,68^ Besides, the X-ray diffraction characterization of pure CS (Fig. 7B) showed a very broad peak at around 2*θ* = 20°, which indicated that the CS was amorphous.^69–71^ This broad peak intensity decreased in the AuNFs-CS pattern, suggesting that the amorphous structure could be reduced while forming the interaction with AuNFs.

### FTIR analysis

**Fig. 8:**
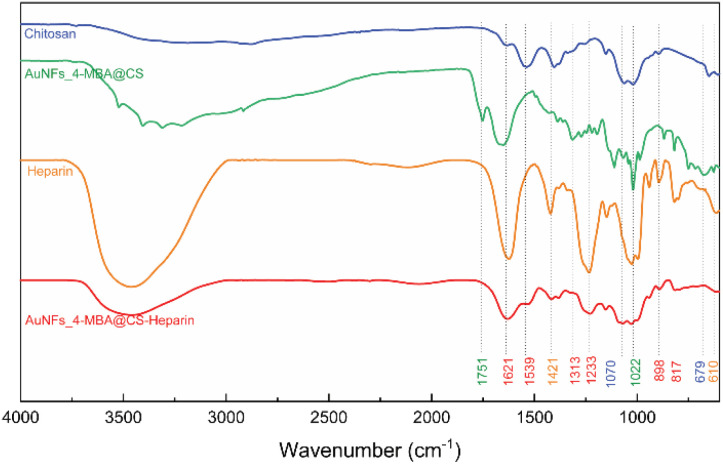
FTIR spectrum of pure chitosan, AuNF-chitosan, pure heparin, and as-prepared AuNF-chitosan-heparin.

The specific interaction between CS functional groups and AuNFs-4-MBA were further determined by FT-IR spectroscopy. The appearance of a peak located at a particular wave number would indicate the existence of a chemical bond. The FTIR spectra and the characteristic peaks of all prepared samples were exhibited in Fig. 8 and Table S1,† respectively. The broad band at the wavenumber of 3447 and 3300 cm^−1^ were assigned to the intermolecular hydrogen-bonded O–H stretching overlapped with N–H stretching vibration in the secondary amides^72,73^ (Fig. 8). It was observed that the absorbance intensity decreased dramatically and shifted to 3523 cm^−1^ in the spectrum of the as-prepared AuNFs-4-MBA@CS sample (Fig. 8). The intensity of C–H stretch observed at 2921 cm^−1^ due to C=O stretching vibration in pure CS becomes much smaller than in the AuNF-4MBA@CS sample and moves to 2916 cm^−1^. The peaks at 1628 cm^−1^ in the pure CS spectrum correspond to C=O stretching vibration of the amide I (–CONH_2_ groups). This peak intensity changed remarkably in the spectrum of AuNFs-4-MBA@CS and shifted to a wavenumber of 1652 cm^−1^, assuming the interaction between the amine group and AuNFs was formed. The peaks assigned to asymmetric C–H stretch bending of the CH_2_ group at 1409 cm^−1^ in pure CS move to 1392 cm^−1^ in the AuNF-4-MBA@CS spectrum. Moreover, the N–H bending vibration peak of amide II at 1539 cm^−1^ in the pure CS spectrum disappeared in the AuNFs-spectrum. The FTIR results clearly revealed that the NH_2_ group of CS has interacted with AuNFs based on the effects on the intensity of specific bands. The pure heparin sample was also compared to the AuNF-4-MBA@CS-heparin to observe their similarities and differences (Fig. 8-curve orange and red). The assignments for the S=O stretch, symmetric, and asymmetric stretch vibrations of carbonyl groups were also observed at 1313, 1233, 1421, and 1621 cm^−1^, respectively. These FTIR results could provide more supporting evidence about the surface of gold nanoparticles and bio-polymer interaction.

### Cytotoxicity of the AuNFs-4-MBA@chitosan

The viability of fibroblast and MCF-7 cells treated with AuNFs-4-MBA@CS at different concentrations from 25 to 100 ppm was evaluated through the SRB assay. As shown in Fig. 10, the formed SER-tags are virtually non-toxic, and the percent of viability was higher than 94%, even with a high concentration of 100 ppm. The testing results ensure that as-prepared AuNFs-4-MBA@chitosan SERS tags are non-toxic and have good biocompatibility, enabling their use for development in biological environment detection. This single-step preparation route is a facile and green method involving no surfactant or organic solvent.

**Fig. 9:**
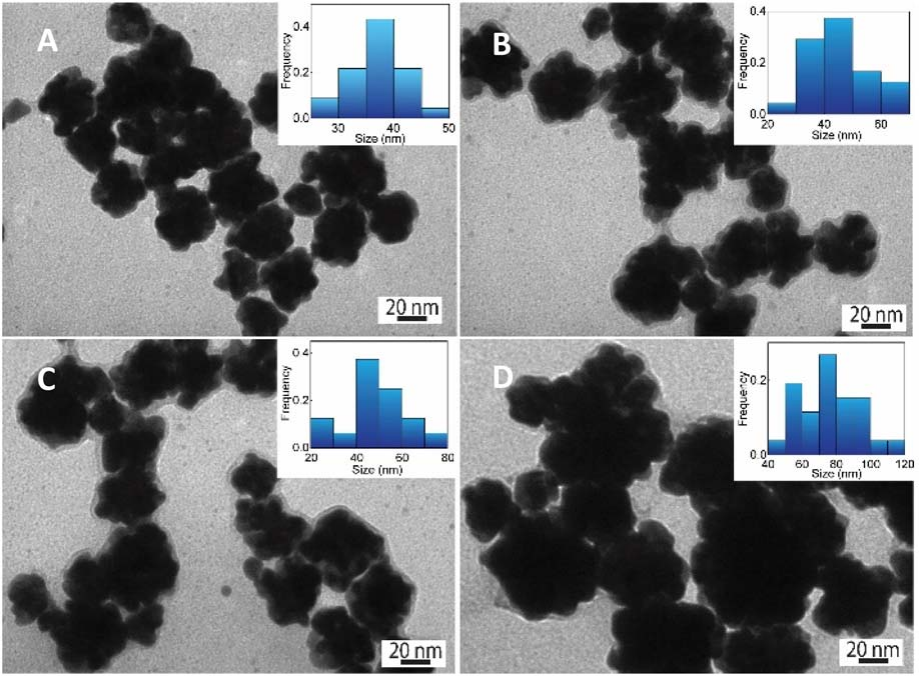
TEM images of AuNFs-4MBA after adding (A) 0.01, (B) 5.0, (C) 30, and (D) 100 ppm of heparin concentrations (scale bars for all, 20 nm).

**Fig. 10:**
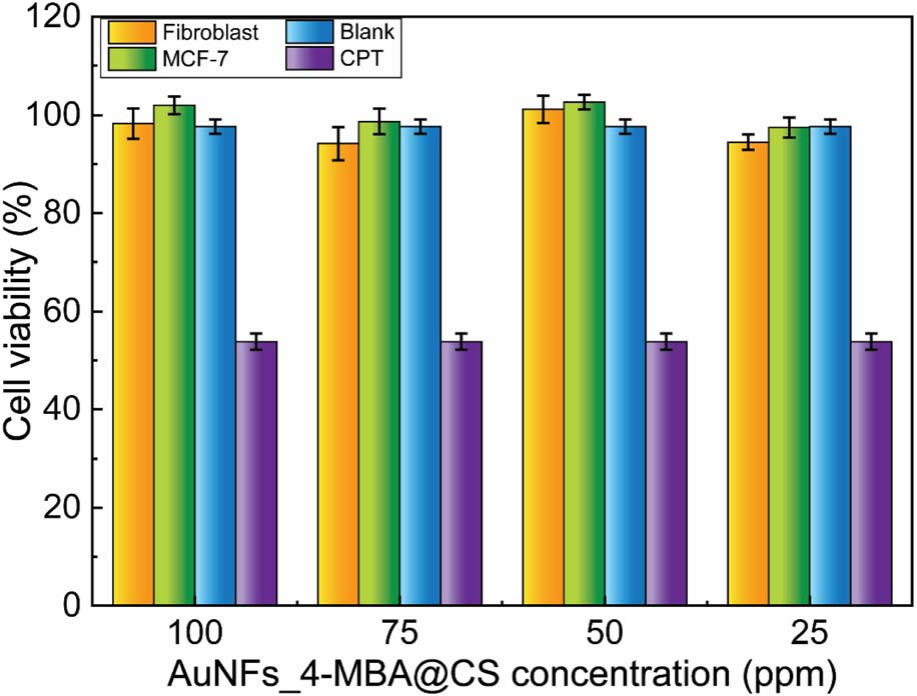
Cell viability study of fibroblast and MCF-7 cells treated with different AuNFs_4-MBA@CS concentrations.

### SERS characterization

SERS effect was known to be more significant when using the Raman molecules reporter in the interstices of the cluster than individual particles.^74^ This study used *p*-MBA as the reporter because of their narrow and distinct spectroscopic signature. Also, the carboxyl function group could interact with the chitosan molecules and assemble them around 4-MBA *via* esterification or amide reaction.^75^ The AuNFs-4-MBA@CS could be induced to aggregate by adding heparin due to the interaction between the positive charge of many amino groups and the negative charge on the analysis molecule heparin.^76^ This aggregation effect can result in the amplifying of 4-MBA signal intensity. Typical SERS spectroscopy of AuNFs-MBA-chitosan and 4-MBA solid was exhibited in (see Fig. S3†). The characteristic Raman peak intensity at 1095 cm^−1^ and 1287 cm^−1^ for the 4-MBA solid sample were significantly increased and shifted to 1075 cm^−1^ and 1360 cm^−1^ for the AuNFs-4-MBA@CS colloidal solution after adding heparin, respectively. The peak at about 2560 cm^−1^ corresponds to the vibrations of the S–H groups in the solid 4-MBA. The strongest band observed at about 1587 cm^−1^ in the SERS spectra of AuNFs-4-MBA@CS colloid is attributed to the *ν*_8a_ aromatic ring vibrations (see Fig. S3†).^77^ The zeta potential of AuNFs-4-MBA@CS and AuNFs-MBA@CS after adding 30 ppm heparin under different pH values was studied to understand the aggregation effect induced by the heparin (see Fig. S4†). With the presence of heparin, the zeta potential values are reversed, indicating that the positive charge on the surface of AuNFs-4-MBA@CS is substituted by the negative charge of heparin molecules under the investigating pH range from 1 to 8. Besides, the self-aggregation of AuNFs-MBA@chitosan after adding heparin was also monitored by TEM images (Fig. 9). As increasing the addition of heparin concentrations (0.01, 5.0, 30, and 100 ppm), the relative space between particles and the self-aggregation state is remarkably changed. UV-Vis spectrum and SEM techniques were also used to study the changes in SPR absorption properties after adding heparin in the range from 0.01 to 100 ppm (see Fig. S5-D†). The maximum SPR peaks shifted to the longer wavelength (from 625 to 670 nm), indicating the change of the AuNFs-4-MBA@chitosan aggregation state. Together with the SEM observation between various heparin concentrations added into the SERS-tags colloids (see Fig. S5† inset A–C), nanoparticles tend to self-aggregate in a cluster. Depending on the amount of heparin, these clusters can gradually increase the number of particles and form more “hot spots”.

**Fig. 11:**
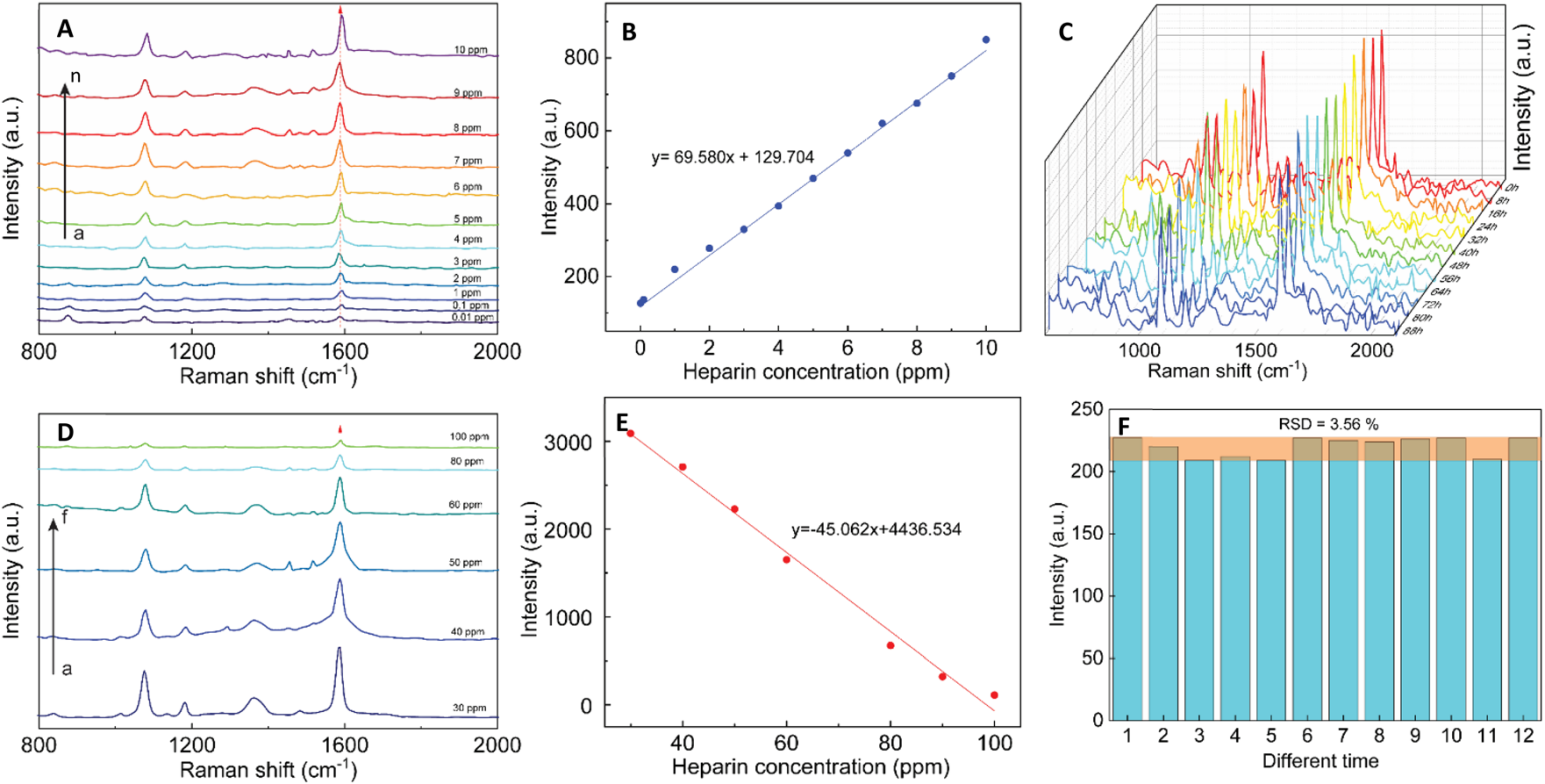
SERS spectra of AuNFs-4-MBA@CS colloids after adding different heparin concentrations: (A) (a–n; 0.01, 0.1, 1.0, 2.0, 3.0, 4.0, 5.0, 6.0, 7.0, 8.0, 9.0, and 10.0 ppm); (C) a serial spectra after adding 1.0 ppm heparin from 12 random times on the colloidal SERS-tags; (D) (a–f; 30, 40, 50, 60, 80, and 100 ppm). (B) and (E) linear plots of Raman intensity at 1587 cm^−1^ against different heparin concentrations. Serial SERS spectra of AuNFs-MBA-4-MBA@CS with heparin concentration of 30 ppm from different times, and (F) Raman vibrational intensities of 4-MBA at 1587 cm^−1^ after adding 1.0 ppm of heparin.

The SERS performance of AuNFs-4-MBA@CS colloids after adding different heparin concentrations is shown in Fig. 11. The Raman peak intensity at 1587 cm^−1^ was enhanced while varying the heparin concentration from 0.01 to 100 ppm. Two different linear correlations exist for two consecutive concentrations range (0.01–10 ppm and 30–100 ppm). Fig. 11B displayed an excellent linear correlation with a high *R*^2^ of 0.998 for the heparin concentration ranging from 0.01 to 10 ppm. The limit of detection (LOD) is 0.054 ppm, and the limit of quantification (LOQ) is 0.17 ppm. The peak intensity of the Raman probe molecules arises concomitantly with the increase of heparin concentrations until it reaches 30 ppm. Conversely, adding more heparin concentration leads to the gradually decreased SERS peak intensity. The relationship between the Raman peak intensity at 1587 cm^−1^ and heparin concentrations added into the SERS tags colloid is illustrated in Fig. S6.† The highest Raman signal intensity is found in the sample added with 30 ppm of heparin concentration. This work's results are slightly similar to a previous report proposed by Zheng *et al.*^78^ However, there are remarkably differences in the concentration points that change signal intensity and the detection windows. The EF value was evaluated using the following equation:

3 EF = (*I*_SERS_ × *C*_b_)/(*I*_b_ × *C*_SERS_)

where *I*_SERS_ and *I*_b_ are the Raman peak intensity of SERS tags-heparin colloidal solution and the solution containing 4-MBA, heparin without using SERS tags. *C*_SERS_ and *C*_b_ are the concentration of the 4-MBA in the SERS tags colloidal solution and the 4-MBA concentration in the bare solution without SERS tags. The EF factor calculated by eqn (3) above is approximately 10^4^ for the Raman peak intensity at 1587 cm^−1^.

The reproducibility and stability of SERS tags are important for their use in bio-environment sensing. In order to investigate the reproducibility, the colloidal SERS tags were diluted into the 1.0 ppm of heparin concentration. Fig. 11C illustrates the Raman intensity of the 4-MBA-labeled peak at 1087 and 1587 cm^−1^ from 12 random times on the colloidal SERS-tags for 1.0 ppm concentration of heparin addition. A slight RSD value of 3.56% at the 1587 cm^−1^ peak revealed that the colloidal SERS tags have good reproducibility (Fig. 11F).

### Selectivity studied

**Fig. 12:**
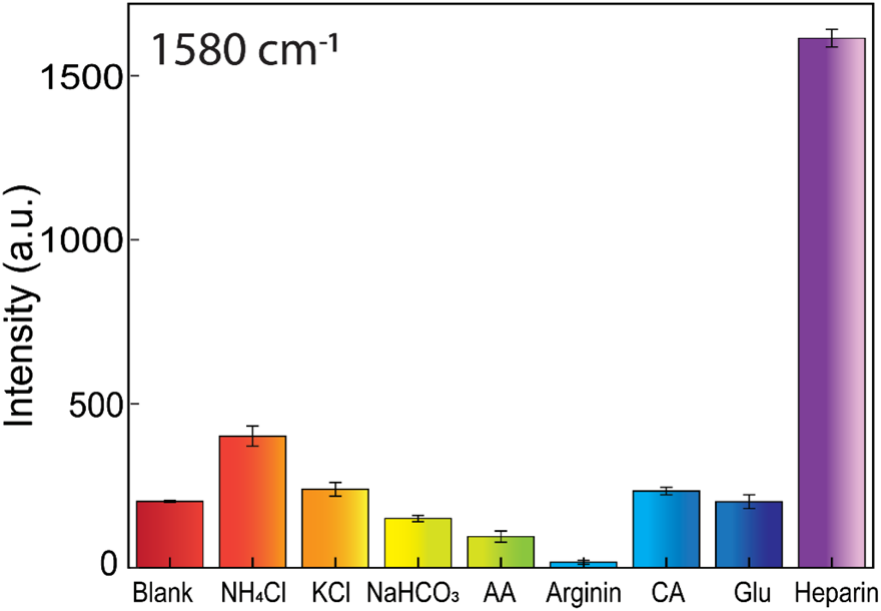
Selectivity study of the AuNFs-4-MBA@chitosan SERS-tag nanoprobe. Comparison of the SERS peak intensity of 4-MBA at 1580 cm^−1^ after adding different substances, involving 50 ppm heparin, 10^−2^ M KCl, 10^−4^ NaHCO_3_, 10^−4^ M NH_4_Cl, 10^−4^ M ascorbic acid (AA), 10^−4^ M arginine, 10^−4^ M citric acid (CA), and 10^−6^ M glucose substances.

Additional comparison experiments between heparin and the other induced aggregation substances, such as NH_4_Cl, KCl, NaHCO_3_, ascorbic acid, arginine, citric acid, and glucose, are conducted to study the SERS-tags colloids' selectivity (Fig. 12). These substances often exist in biological environments. They are also capable of causing flocculation of AuNFs-4-MBA@CS system. The testing concentration of ascorbic acid, arginine, citric acid, NaHCO_3_, and NH_4_Cl was 10^−4^ M. The KCl and glucose concentrations are 10^−2^ M and 10^−6^ M, respectively, while the heparin concentration is 50 ppm. The obtained results revealed that the investigated substances only caused a minor effect on the amplifying SERS intensity peak at 1580 cm^−1^ of the 4-MBA compared with heparin (Fig. 12). The ability to enhance the Raman 4-MBA signal is very specific when adding heparin according to the strong characteristic peak (see Fig S7†). These substances interaction with the positive charge of AuNFs-4-MBA@chitosan may not be as effective as the negatively charged heparin, which possesses carboxylate per repeat unit. All the obtained results showed that the development of AuNFs-4-MBA@chitosan SERS tags colloid has the ideal selectivity and can be potentially utilized in biological fluids.

## Conclusions

The SERS-tags colloids have been successfully prepared through a single-step procedure. The AuNFs-4-MBA@CS includes three vital components: gold nanoflower with controlled size, Raman probe molecules for detection signature, and chitosan encapsulation for enhancing protection. The formation and properties of the SERS tags were further confirmed by UV-Vis, SEM, TEM, HRTEM, FTIR, SAED, and X-ray diffraction patterns analyzed. The cytotoxicity testing has demonstrated that the obtained SERS-tags colloids are non-toxic on the fibroblast and MCF-7 with a high percent cell viability above 94.0% even at high testing concentrations. The positive charged AuNFs-4-MBA@chitosan particles showed an excellent amplification of the 4-MBA signal. The trace amount of heparin could be detected with a LOD of 0.054 ppm, a LOQ of 0.17 ppm, and a concentration range of 0.01 to 100 ppm. This strategy also has good reproducibility (RSD value of 3.56%) and high selectivity compared with electrolytes ions, organic acids, glucose, and amino acids. The AuNFs-4-MBA@CS colloidal SERS tags would be an improvement for various applications in biological environment detection because of their non-toxicity and minimal interference of non-target biomolecules.

## Abbreviations

AuNFs: Gold nanoflowers
4-MBA: 4-Mercapto benzoic acid
DMEM: Dulbecco's modified Eagle's medium
FTIR: Fourier-transform infrared
PBS: Phosphate-buffered saline
TEM: Transmission electron microscopy
CS: Chitosan

## Author contributions

Conceptualization, T. A. N. and K. Q. V.; methodology and analysis, T. N. H. L., I. P. investigation, T. A. N., K. Q. V. writing-original draft preparation T. A. N., K. Q. V.; writing-review and editing I. P., and K. Q. V.; supervision, K. Q. V. All author reviewed the manuscript.

## Conflicts of interest

There are no conflicts to declare.

## Supplementary Material

RA-012-D2RA06528B-s001
