# Supplementary material for: Single-step controlled synthesis of flower-like gold nanoparticles stabilized by chitosan for sensitive detection of heparin using a surface-enhanced Raman scattering method
Source: RSC Adv. 2022 Dec 6;12(54):34831–42. doi: 10.1039/d2ra06528b (PMC9724128; doi:10.1039/d2ra06528b)
Supplement: RA-012-D2RA06528B-s001 [file RA-012-D2RA06528B-s001.pdf]

Electronic Supplementary Material (ESI) for RSC Advances.
This journal is © The Royal Society of Chemistry 2022
Please do not adjust margins

RSC Advances

SUPPLEMENTARY INFORMATION

SINGLE-STEP CONTROLLED SYNTHESIS OF FLOWER-LIKE GOLD NANOPARTICLES STABILIZED BY CHITOSAN FOR SENSITIVE DETECTION OF HEPARIN USING SURFACE ENHANCED RAMAN SCATTERING METHOD

Thu Anh Nguyen [a], Anh Nguyen Kim Do [a], Tien Nu Hoang Lo [b], In Park*[b,c], and Khuong Quoc Vo*[a]

[a.] *Faculty of Chemistry, University of Science, Vietnam National University - Ho Chi Minh City, 227 Nguyen Van Cu Street, Ward 4, District 5, Ho Chi Minh City 70000, Vietnam.*
[b.] *Research Institute of Clean Manufacturing System, Korea Institute of Industrial Technology (KITECH), 89 Yangdaegiro-Gil, Ipjang-myeon, Cheonan 31056, South Korea.*
[c.] *KITECH school, University of Science and Technology (UST), 176 Gajeong-dong, Yuseong-gu, Daejeon 34113, South Korea.*
**Corresponding authors*
Email addresses: vqkhuong@hcmus.edu.vn (Khuong Quoc Vo),
inpark@kitech.re.kr (In Park)

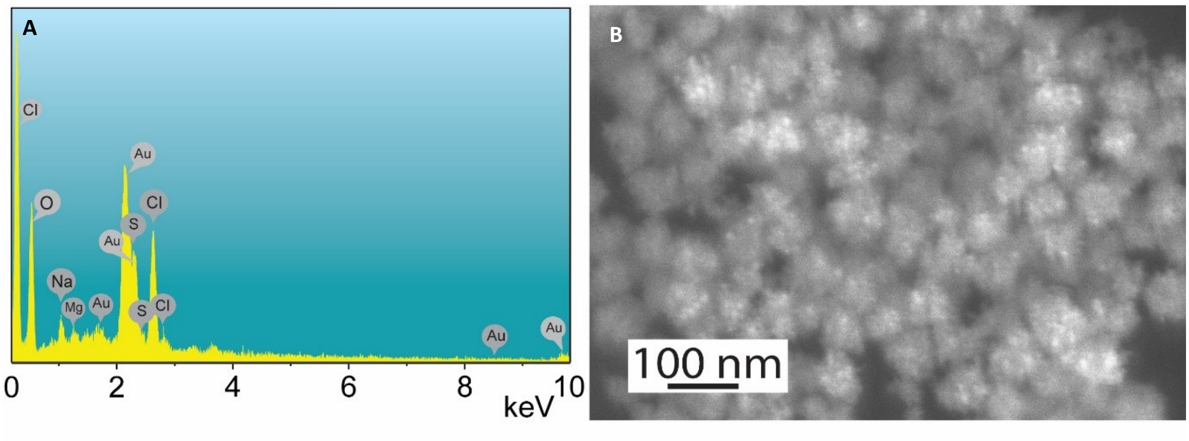

Fig. S1 (A) EDS spectroscopy and (B) corresponding SEM images of formed AuNFs-4-MBA@chitosan (scale bar 100 nm).

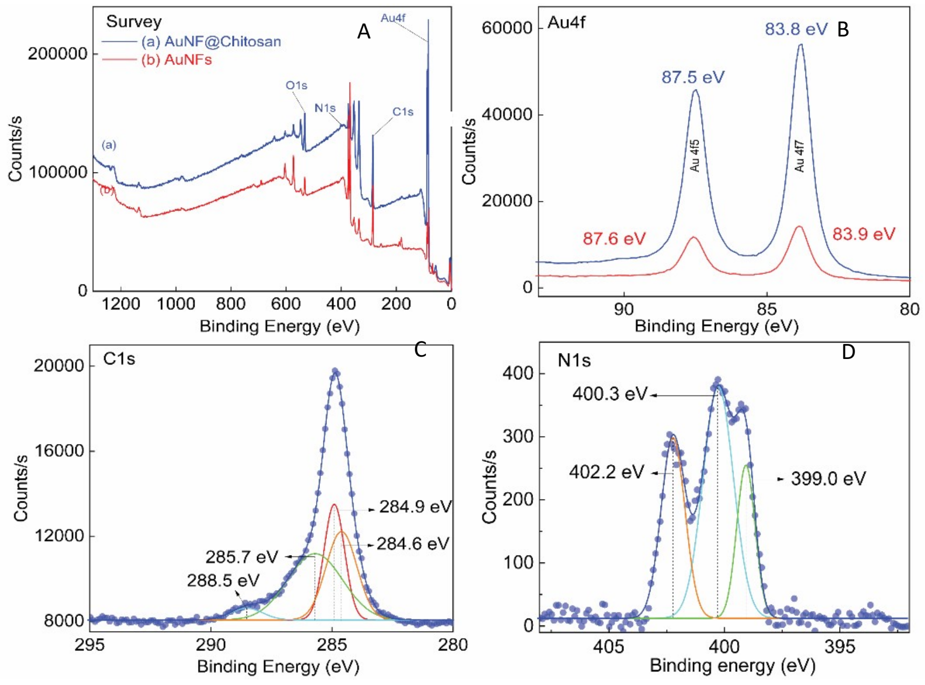

Fig S2. (A) XPS survey spectra of the AuNFs stabilized with chitosan, high-resolution XPS spectra of (B) Au4f, (C) C1s, and (D) N1s.

Please do not adjust margins

Table S1. FTIR band positions of AuNFs-chitosan, pure chitosan, pure heparin, and AuNF-chitosan-heparin.

Wavenumber (cm^{-1})				Assignment	Ref
**AuNFs-4-MBA@CS**	**Pure CS**	**Pure HEP**	**AuNFs-4-MBA@CS after adding HEP**		
3523	3447			O-H overlapped with N-H stretch	1, 2
3311					
2916	2921			C-H stretch	1, 3
1753	1700			Amide II stretch	
1628	1657			C=O stretching in amide I acetyl amino in heparin	1, 3
		1620	1621		4
	1539		1539	N-H stretch (amide II)	1, 2, 5
1392	1409			Asymmetric C-H stretch bending of CH_2 group	1
	1369			Amide III	
	1319	1313		C-O stretch, S=O	6
		1233	1233	N-sulphonate	7-9
1112				C-H symmetrical bending	10
1018	1066			Skeletal vibration C-O bridge (glucosamine)	1, 10
869		890		OH in-plane bending	10
				Coupling of C-O-S and ring C-O-C	11

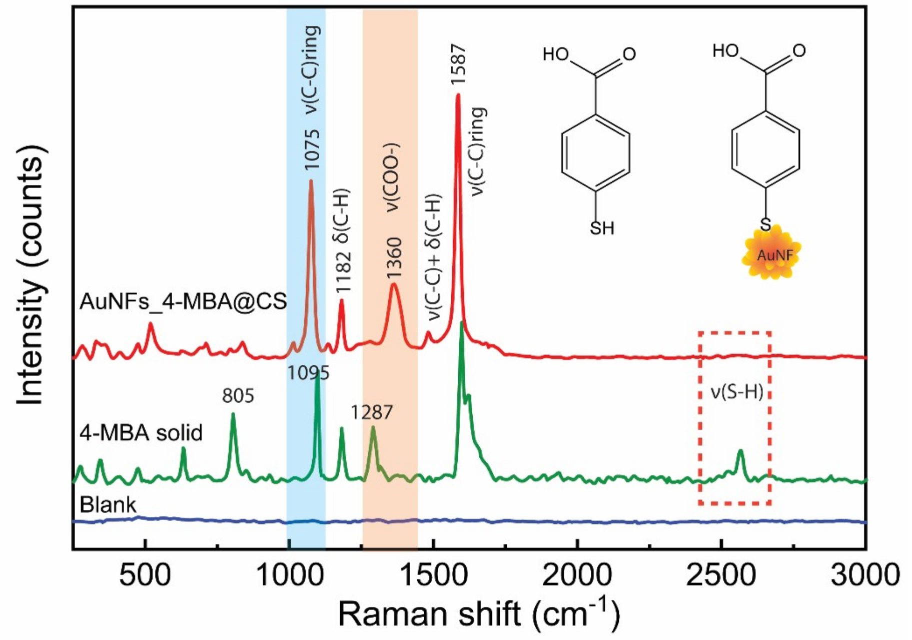

Fig S3. Compared SERS spectroscopy of (A) 4-MBA solid and (B) AuNFs-MBA@CS after adding chitosan.

This journal is © The Royal Society of Chemistry 20xx

Please do not adjust margins

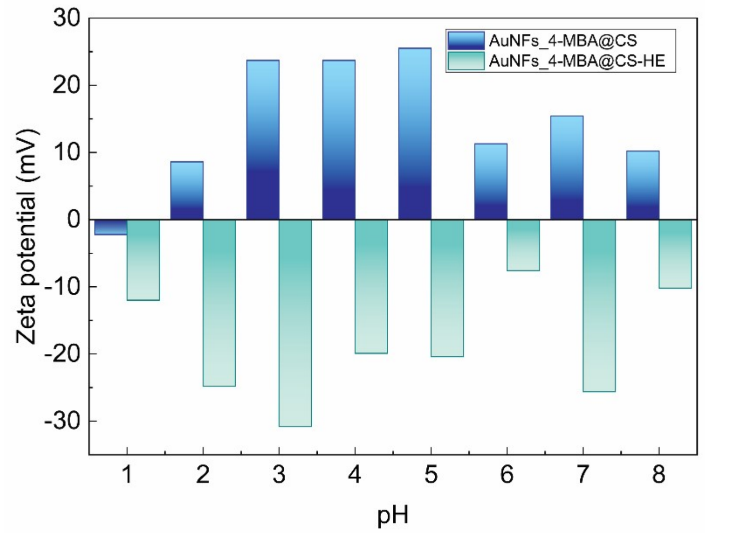

Fig S4. Zeta potential of AuNFs-chitosan (blue column), and AuNFs-4-MBA@chitosan-heparin (green-column).

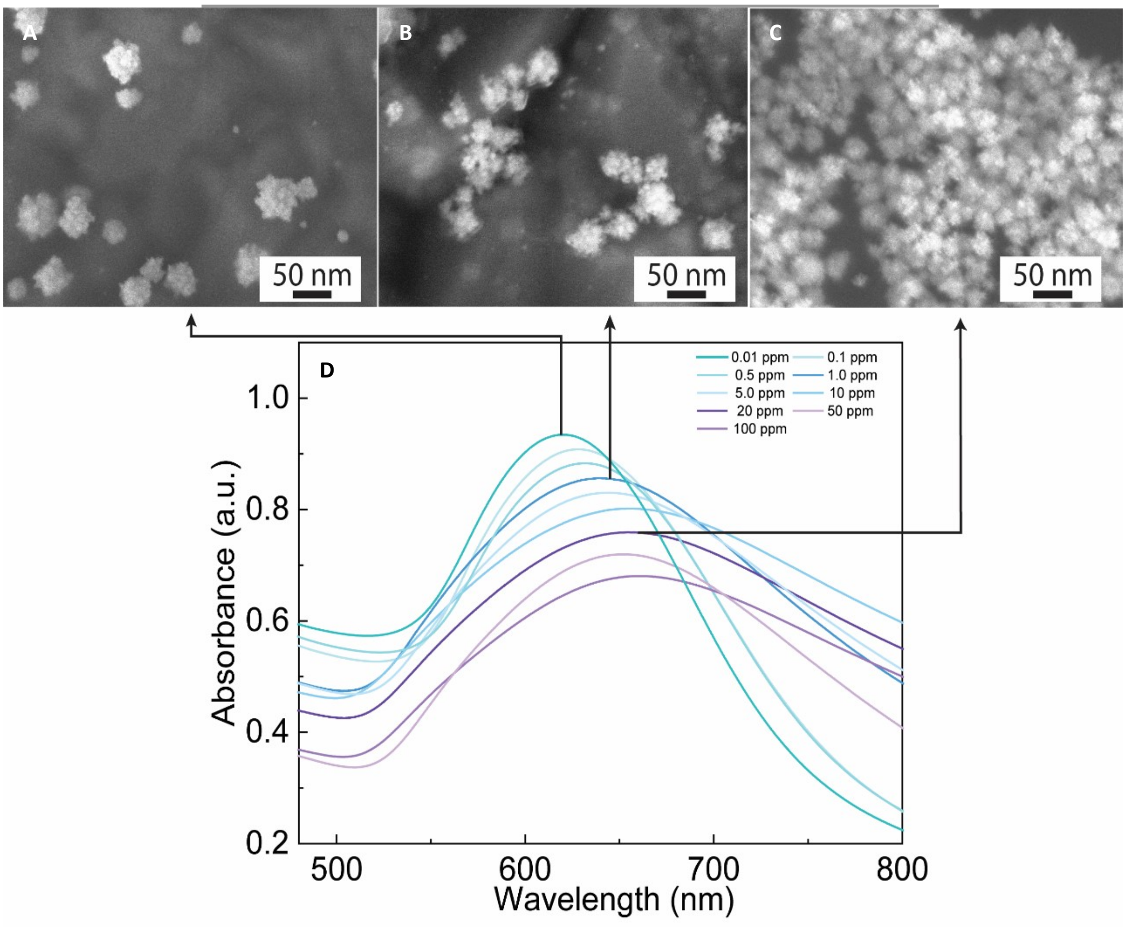

Fig S5. (A), (B), (C) SEM images of colloidal SERS tags after adding 0.01, 1.0, and 20 ppm concentration of heparin, and (D) UV-Vis spectrum of AuNFs-4-MBA@Chitosan colloids added with different heparin concentrations ranging from 0.01 to 100 ppm

Please do not adjust margins

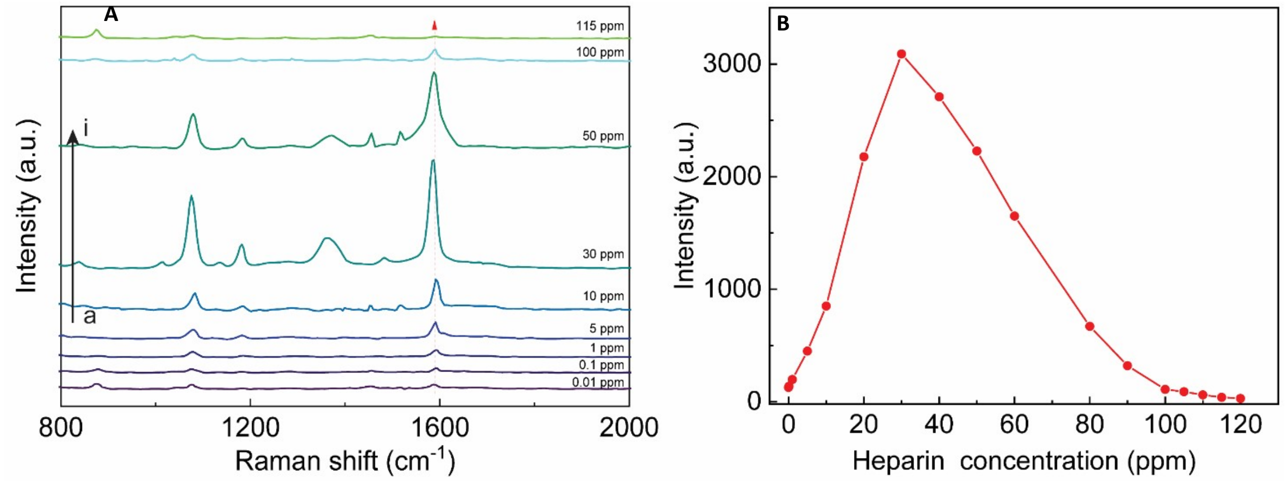

Fig S6. (A) SERS spectra of AuNFs-4-MBA@CS colloidal solution studied with different heparin concentrations (a-i: 0.01, 0.1, 1.0, 5.0, 10, 30, 50, 100, and 115 ppm); (B) Intensity change of Raman peak at 1587 cm^{-1*} while changing the heparin concentration.

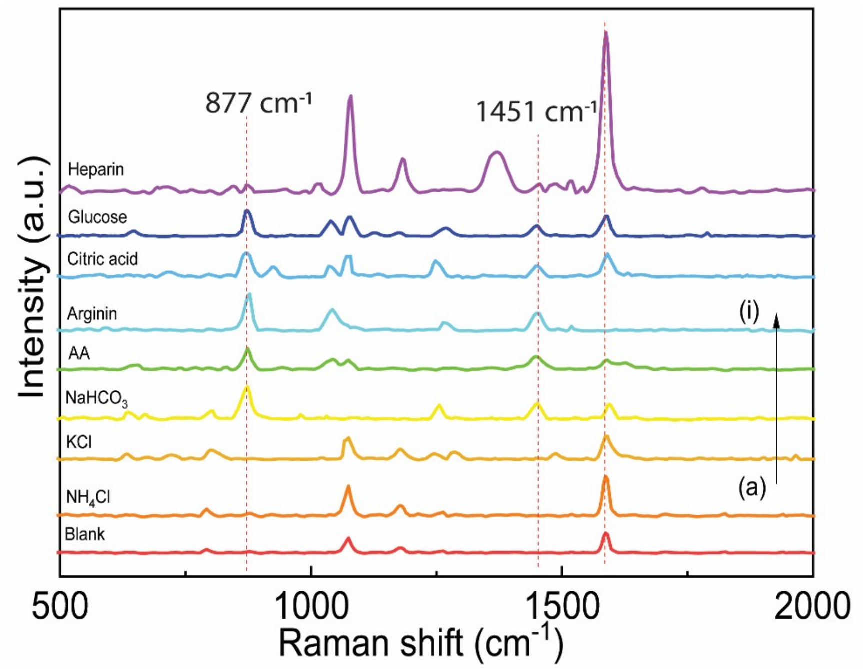

Figure S7. Compared SERS spectra of AuNFs-4-MBA@CS colloids after adding various substances (a-i: glucose, citric acid, arginine, ascorbic acid, $NaHCO_3$, KCl, NH_4Cl, and heparin) for studying the selectivity. The blank sample was investigated on the AuNFs-4-MBA@chitosan colloid without using any induced-aggregation substance.

This journal is © The Royal Society of Chemistry 20xx

Please do not adjust margins

Reference

1. S. Kumar, J. Koh, H. Kim, M. K. Gupta and P. K. Dutta, *International journal of biological macromolecules*, 2012, **50**, 493-502.
2. S. Kumar and J. Koh, *Int J Mol Sci*, 2012, **13**, 6102-6116.
3. J. E. dos Santos, E. R. Dockal and É. T. Cavalheiro, *Carbohydrate polymers*
2005, **60**, 277-282.
4. R. Saravanan, S. Vairamani and A. Shanmugam, *Preparative biochemistry & biotechnology*, 2010, **40**, 305-315.
5. Q. K. Vo, D. D. Phung, Q. N. Vo Nguyen, H. Hoang Thi, N. H. Nguyen Thi, P. P. Nguyen Thi, L. G. Bach and L. Van Tan, 2019, **7**, 873.
6. A. Devlin, L. Mauri, M. Guerrini, E. Yates and M. Skidmore, *The use of ATR-FTIR spectroscopy to characterise crude heparin samples by composition and structural features*, 2019.
7. M. J. Harris and J. R. Turvey, *Carbohydrate Research*, 1970, **15**, 51-56.
8. D. Grant, C. Moffat, W. Long and F. Williamson, *Biochemical Society Transactions*, 1989, **17**, 498-500.
9. D. Grant, C. Moffat, W. Long and F. Williamson, *Biochemical Society Transactions*, 1989, **17**, 500-501.
10. s. Sashikala and S. Shafi, *Der Pharmacia Lettre*, 2014, **6**, 90-97.
11. F. Cabassi, B. Casu and A. S. Perlin, *Carbohydrate Research*, 1978, **63**, 1-11.

[a] *Faculty of Chemistry, University of Science, Vietnam National University - Ho Chi Minh City, 227 Nguyen Van Cu Street, Ward 4, District 5, Ho Chi Minh City 70000, Vietnam.*
[b] *Research Institute of Clean Manufacturing System, Korea Institute of Industrial Technology (KITECH), 89 Yangdaegiro-Gil, Ipjang-myeon, Cheonan 31056, South Korea.*
[c] *KITECH school, University of Science and Technology (UST), 176 Gajeong-dong, Yuseong-gu, Daejeon 34113, South Korea.*
**Corresponding authors*
Email addresses: vqkhuong@hcmus.edu.vn (Khuong Quoc Vo),
inpark@kitech.re.kr (In Park)

Electronic Supplementary Information (ESI) available: [details of any supplementary information available should be included here]. See DOI: 10.1039/x0xx00000x

This journal is © The Royal Society of Chemistry 20xx
